# Campbell title registrations to date—August 2022

**DOI:** 10.1002/cl2.1275

**Published:** 2022-09-13

Details of new titles for systematic reviews or evidence and gap maps that have been accepted by the Editor of a Campbell Coordinating Group are published in each issue of the journal. If you would like to receive a copy of the approved title registration form, please send an email to the Managing Editor of the relevant Coordinating Group.

## AGEING

The effectiveness of sensory interventions targeted at improving occupational outcomes, quality of life, well‐being, and behavioural and psychological symptoms for older adults living with dementia: A systematic review and meta‐analysis

Nikki Tulliani, Caroline Mills, Nicole Peel, Paul Fahey, Karen Liu

20 July 2022

## SOCIAL WELFARE

Resilience measures and their psychometric properties: A systematic review

Donncha Hanna, Liam O'Hare, Laura Dunne, Richard Lombard‐Vance, Sarah Miller

8 August 2022

The effectiveness of skills training to increase employment amongst those experiencing and at risk of homelessness

Zijun Li, Yanfei Li, Ke Guo, Mina Ma, Minyan Yang, Kehu Yang

10 July 2022

Participation in organised sport to improve and prevent adverse developmental trajectories of at‐risk youth: A systematic review

Trine Filges, Mette Verner, Else Ladekjær, Elizabeth Bengtsen

1 June 2022

